# Outcomes of cemented taper slip versus composite beam femoral stems in total hip arthroplasty: A systematic review and meta-analysis

**DOI:** 10.5339/qmj.2025.20

**Published:** 2025-02-04

**Authors:** Loay A. Salman, Harman Khatkar, Jawad Derbas, Mohammed F.A. Radi, Jehad Feras AlSamhori, Wael Al-Atout, Osama Z. Alzoubi, Ghalib Ahmed

**Affiliations:** ^1^Department of Orthopedic Surgery, Hamad General Hospital, Hamad Medical Corporation, Doha, Qatar; ^2^Royal London hospital, Whitechapel, London, UK; ^3^Faculty of Medicine, University of Jordan, Amman, Jordan; ^4^Department of Orthopedic Surgery, Speciality Hospital, Amman, Jordan *Correspondence: Loay A. Salman. Email: loayasalman@gmail.com

**Keywords:** Total hip arthroplasty, Charnley, Exeter, composite beam, taper slip

## Abstract

**Objective:**

The aim of this systematic review and meta-analysis was to compare the outcomes of cemented collared composite beam (CB) and collarless taper slip (TS) femoral stems in total hip arthroplasty (THA).

**Methods:**

Four databases were searched from inception to August 2023 for original studies that compared the outcomes of cemented CB and TS femoral stems following THA. The primary outcome was aseptic loosening, and the secondary outcomes were periprosthetic fractures, instability, dislocation, revision, survivorship, and periprosthetic joint infection (PJI). This review was conducted in accordance with PRISMA (Preferred Reporting Items for Systematic Reviews and Meta-Analyses) guidelines.

**Results:**

A total of 11 studies with 730,769 hips were included, with a mean follow-up period of 8.48 ± 6.07 years and average MINORS (Methodological Index for Non-Randomized Studies) score of 17.36 ± 1.86. There was a statistically significant difference in aseptic loosening (OR 0.28, 95% CI 0.19–0.43, *p* < 0.001) and PJI rate (OR 0.61, 95% CI 0.53–0.71, *p* < 0.001) between the CB and TS groups in favor of the latter. However, periprosthetic fracture, revision rate, survivorship, instability, and dislocation were similar in both groups (*p*  =  NS).

**Conclusion:**

This study showed a significantly higher aseptic loosening and PJI in the CB group compared to the TS stem type. However, other outcomes were comparable. Due to confounding effects, these results must be interpreted in context.

## Introduction

Total hip arthroplasty (THA) is a commonly performed orthopedic surgery, with more than 500,000 and 95,677 THA performed annually in the USA and UK, respectively.^1,2^ Understanding the underlying biomechanical and inflammatory nature of osteoarthritis (OA) is crucial,^3^ with THA considered as the treatment of choice for end-stage hip osteoarthritis.^4^

There are various designs and fixation methods for total hip prosthesis. In cemented THA, the design of the femoral stem follows one of two biomechanical principles to ensure stability in the femoral canal under constant axial load. The first design follows the “force-closed” or “polished-taper slip” (TS) principle, while the second uses the “shape-closed” or “composite beam” (CB) principle.^5^ The most popular example on the CB is the original Charnley (DePuy International) stem, while examples of TS stem designs include Exeter V40 (Stryker), CPT stem (Zimmer), and C-Stem (DePuy International).

Several studies have shown equivalent results in both types of stems with excellent overall implant survivorship and different failure modes in both groups. Therefore, the choice between a CB and a tapered slip stem remains controversial.^6,7^ The Charnley stem provided excellent outcomes with long survival rates due to its low friction properties.^8^ The Exeter and Exeter V40 stems also showed favorable long-term survival rates.^9,10^ However, limitations included low statistical power and short duration of the follow-up period. Therefore, robust evidence is required to thoroughly compare outcomes between the two groups.

The aim of this study was to compare the clinical outcomes, including aseptic loosening, dislocation, instability, periprosthetic fracture, periprosthetic joint infection (PJI), revision rate, and survivorship between cemented CB and TS femoral stems. We hypothesize that there is no significant difference in outcomes and complication rates between the two groups.

## Methods

This systematic review was conducted in accordance with the Preferred Reporting Items for Systematic Reviews and Meta-Analyses (PRISMA) guidelines.^11^ The protocol was pre-registered in the International Prospective Register of Systematic Reviews (PROSPERO) under the registration number CRD42023458881.

### Search strategy

Four online databases (Ovid MEDLINE, Embase, Web of Science and Cochrane Library databases) were searched from inception to August 1, 2023 to identify all studies that compared collared CB versus collarless TS femoral stems in THA. The following keywords and their derivatives were included: Taper Slip OR Exeter AND Composite Beam OR Charnley AND Femoral Stem AND Cemented AND Total hip arthroplasty.

### Eligibility criteria

Studies were considered eligible if they met the following criteria: (1) comparison of any of the clinical outcomes of interest between the CB and TS groups, (2) all types of cemented femoral stems (regardless of the design or manufacturer), (3) primary THA, and (4) published in the English language.

Exclusion criteria included the following: (1) failure to compare outcomes of both femoral stem types, (2) cementless or revision THA, (3) studies with incomplete or unextractable data for review, and (4) review articles and preclinical and case reports.

### Study screening

Two authors conducted the screening process independently and blindly by screening the titles and abstracts of the retrieved articles. Full-text review was performed for studies that met the pre-specified eligibility criteria. Any disagreements between the two authors were resolved by a third senior author. The references of the included articles were manually searched to ensure that all relevant studies were included.

### Data extraction

Two authors independently extracted the data from the included articles. The following data were collected: study characteristics, patient demographics, study year, design and location, mean patient age, number of participants and hips, age, type of total hip replacement, type of femoral stem, manufacturer, type of prosthesis and bearings used, mean follow-up period, number of revisions, survivorship, complications, mode of revisions, patient-reported outcomes (if any), statistical tests used, and the conclusion of each study.

### Quality assessment

Two authors conducted the methodological quality assessment blindly and independently using the methodological index for non-randomized studies (MINORS) criteria.^12^ According to the MINORS criteria, comparative and non-comparative studies can achieve a maximum score of 24 and 16, respectively. Comparative studies are graded as very low quality (0–6), low quality (7–10), fair quality (11–16), good quality (16–20), and high quality ( ≥ 20). Non-comparative studies are grade as very low quality (0–4), low quality (5–7), fair quality (8–12), and high quality ( ≥ 13).^12^

## Results

### Study selection

**Figure 1.**
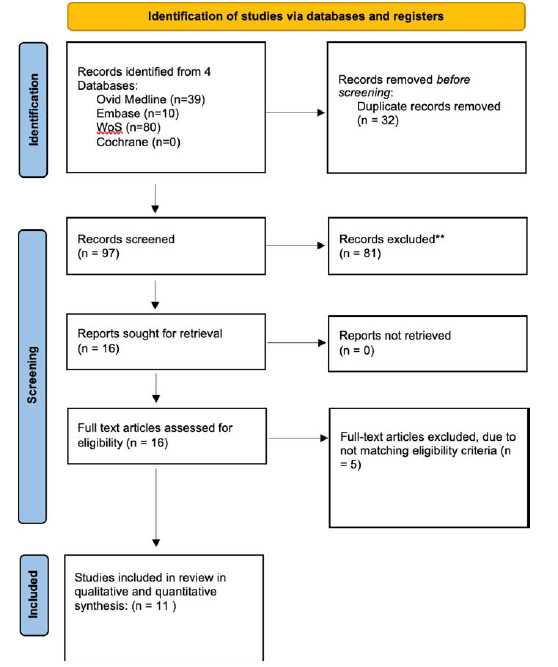
PRISMA flow diagram of record identification, screening, and selection in meta-analysis.

The search across multiple databases yielded a total of 129 articles. After removing 32 duplicates, 97 records underwent initial screening based on their titles and abstracts, excluding 81 records. Ultimately, 16 papers were eligible for full-text review. As a result, 11 studies met the eligibility requirements and were included in both qualitative and quantitative analyses. The process is shown in Figure 1 using the PRISMA flowchart.

### Characteristics of the included studies

A total of 11 research papers were included. These studies were published between 1997 and 2023 and originated primarily from various regions, including the UK (three articles), the USA (two articles), Norway (two articles), and Scotland, Finland, the Netherlands, and Ireland (one article each). Of the 11 included studies, 10 were retrospective and one was prospective. The total number of hips examined across the studies was 730,769. The mean follow-up duration was 8.48 ± 6.07 years. Table 1 summarizes the characteristics of the studies.

### Quality assessment (level of evidence and risk of bias)

We evaluated the methodological quality of the included studies using the MINORS criteria, reflecting how well each study adhered to predefined methodological quality standards. Higher scores indicate more robust methodological rigor. The MINORS criteria scores of the included studies ranged from 14 to 20, with an average of 17.36 ± 1.86 (out of 24), indicating an overall low risk of bias. A summary of the quality assessment according to the MINORS criteria is presented in Table 2.

### Aseptic loosening

**Figure 2.**
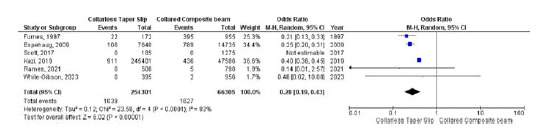
Forest plot comparison of aseptic loosening between the CTS and CCB groups. CI: confidence interval.

Six studies compared the occurrence of aseptic loosening between the collarless TS and collared CB groups. The odds of aseptic loosening were significantly lower in the collarless TS group than in the collared CB group (OR 0.28, 95% CI 0.19–0.43, *p* < 0.001). Due to significant heterogeneity (*I* ^2^ = 83%, *p* < 0.001), a random-effects model was used (Figure 2).

### Dislocation

**Figure 3.**
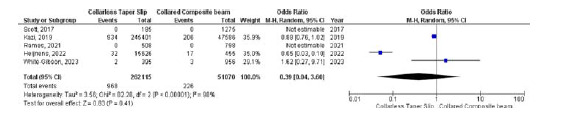
Forest plot comparison of dislocation rates between the CTS and CCB groups. CI: confidence interval.

Five studies were included to assess any differences in dislocation between the collarless TS and collared CB arms. Due to the high heterogeneity (*I* ^2^ = 98%, *p* < 0.001), a random-effects model was used. No significant differences were found in dislocation rates between the two groups (OR 0.39, 95% CI 0.04–3.60, *p* < 0.001) (Figure 3).

### Recurrent instability

**Figure 4.**
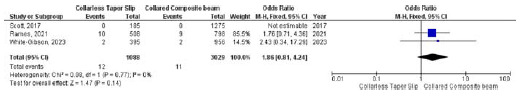
Forest plot comparison of instability between the CTS and CCB groups. CI: confidence interval.

Three studies were included to examine the differences in the occurrence of instability between collarless TS and collared CB stems. No significant differences in instability were observed between the two groups (OR 1.86, 95% CI 0.04–4.24, *p* = 0.14). The heterogeneity was low (*I* ^2^ = 0.0%, *p* = 0.77) (Figure 4).

### Periprosthetic fracture

**Figure 5.**
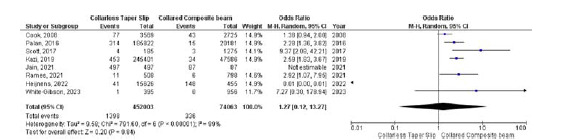
Forest plot comparison of periprosthetic fracture between the CTS and CCB groups. CI: confidence interval.

Eight studies evaluated the odds of periprosthetic fracture occurring between collarless TS and collared CB groups, with no significant differences between the two groups (OR 1.27, 95% CI 0.12–13.27, *p* = 0.84). However, there was significant heterogeneity (*I* ^2^ = 99%, *p* < 0.001), leading to the use of a random-effects model (Figure 5).

### Periprosthetic joint infection

**Figure 6.**
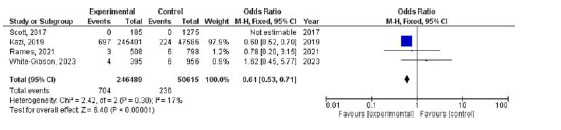
Forest plot comparison of periprosthetic joint infection between the CTS and CCB groups. CI: confidence interval.

Four studies were included to examine the differences in the occurrence of PJI between the two femoral stem types. The use of a collarless TS was associated with a significantly lower likelihood of PJI compared to a collared CB (OR 0.61, 95% CI 0.53–0.71, *p* < 0.001). The heterogeneity was low (*I* ^2^ = 17%, *p* = 0.30) (Figure 6).

### Revision rate

**Figure 7.**
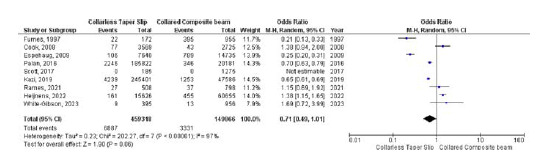
Forest plot comparison of revision rates between the CTS and CCB groups. CI: confidence interval.

Nine studies were analyzed to assess the differences in revision rates between the two groups. No significant differences were observed between the two groups (OR 0.71, 95% CI 0.49–1.01, *p* = 0.06). High heterogeneity was found (*I* ^2^ = 97%, *p* < 0.001) (Figure 7).

### Implant survivorship

**Figure 8.**
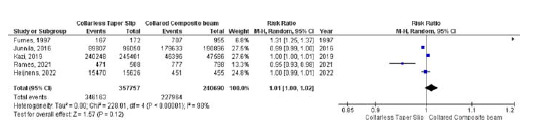
Forest plot comparison of implant survival rates between the CTS and CCB groups. CI: confidence interval.

Five studies reported implant survival rates. No difference in the implant survival rates was found between collarless TS and collared CB stems (RR 1.01, 95% CI 1.00–1.02, *p* = 0.12) (*I* ^2^ = 98%, *p* < 0.001) (Figure 8).

### Publication bias

The Egger bias test revealed insignificant publication bias in the assessment of aseptic loosening (*p* = 0.5701), dislocation (*p* = 0.2393), instability (*p* = 0.4860), periprosthetic fracture (*p* = 0.2080), PJI (*p* = 0.0756), revision (*p* = 0.1726), and implant survivorship (*p* = 0.2609).

## Discussion

This systematic review represents the most comprehensive evaluation of the two types of cemented stems available in the current literature, with the use of registry-level data allowing for a high number of included cases. The use of registry data facilitates comparison and detection of subtle statistical signals within the dataset. However, understanding the clinical relevance of such signals sometimes remains unclear.^6,23,24^

The main finding of this analysis supports the notion that CB stems are at higher risk of aseptic loosening than TS stems. Although no definitive mechanism for this can be theorized, CB stems are considered to be less involving in terms of surgical technique, relying on an adequately thick cement mantle that binds the bone to the cement and the cement to the stem.^5,6,22^ In order for the stem to function as a CB construct, a perfect bonding between the implant and the cement mantle must always be maintained.^6,22,25^ If a perfect bond is maintained, shear forces at the cement–bone interface promotes the production of third-body wear particles, thereby accelerating wear particle-induced periprosthetic osteolysis.^25^ Furthermore, the study by Kärrholm et al. demonstrated that CB stems exhibit subsidence, thus negating the underpinning design philosophy of a CB-type stem.^26,27^ The need for meticulous cementing when using CB stems may increase the propensity for loosening, but there are no clinical studies that support this notion. To fully evaluate this finding, implant retrieval-based studies examining the mechanism of CB stem failure are needed.

The associated difference in the rate of aseptic loosening between stem types cannot be easily explained by biomechanical principles, as the underlying mechanism currently remains unclear. Focused research is required to further evaluate this discrepancy.

The consensus in the literature supports the notion that TS stems have a higher rate of periprosthetic fracture than CB stems.^18,28–33^ This is due to a purported mechanism whereby the polished stem does not remain fixed to the cement mantle, as the stem is designed to subside in a controlled manner within the mantle during loading.^19^ This then leads to the formation of hoop stresses and subsequent long spiral fracture patterns.^18,19,30^ The results of this review did not support this conclusion, with no significant differences being found. This finding reinforces the notion that concern about TS stems and periprosthetic fractures may currently be unfounded, with further basic scientific work required to evaluate whether the fracture mechanism in TS stems conforms to the current hypothesis.

Equivalence between the parameters of dislocation rate, instability, all-cause revision, and survivorship was determined from the findings of this review. This supports the notion that either the CB or TS stem can be considered a viable long-term option, with both guiding principles of design providing a reliable, repeatable femoral stem for patients.The work of this study has elicited a finding that has not been previously reported in the current literature. The PJI rate was significantly lower in TS stems than in CB stems. Given the current understanding of the philosophy of stem design, implantation, and implant performance, no clear explanation for this finding can be definitively hypothesized. Principally, PJI is multifactorial, with host, surgical, and implant factors all contributing to an eventual diagnosis. Isolating the role of stem choice within this area may improve the overall understanding of PJI etiology.^34,35^ The influence of stem design does not appear to be a current area of PJI-related research. However, given the possible discrepancy in infection rates, further work may be required to evaluate this difference.

This review lacked specific data on specific stems, with a focus on the philosophy of stem design. For each individual TS or CB stem, the design philosophy, stem dynamics, and implantation technique vary between manufacturers. Although the design philosophy is useful at the basic science level, it should be evaluated along with specific stem designs. This could enable a more crystallized performance of specific stems in the future. To date, two randomized trials have been performed, directly comparing the Charnley stem with double- and triple-tapered TS designs (Exeter and C-stem) and both studies demonstrating equivalence between stem types at 2 and 5 years.^1,5,25^ It clear that future analysis building on the work of this study should include appropriate analysis of individual stem types to more clearly define any differences between specific CB/TS stem types. This should be performed at the registry level to ensure a sufficiently significant sample size and to correctly identify any discrepancies between stem types.

Additionally, beyond the design of the femoral stem, the outcomes and long-term performance of implants in THA are influenced by various other factors. These include surgical elements such as the cementing technique and specific surgical approach, as well as patient-related factors such as age, activity level, and the underlying reason for undergoing THA.^36,37^ These factors have been associated with implant survivorship and the overall risk of complications, as well as can play a confounding role in outcome assessment.

## Conclusion

This study found a significantly higher incidence of aseptic loosening and PJI in the CB stem type compared to the TS group. However, no differences were observed between the groups in terms of periprosthetic fracture, revision rate, survivorship, instability, or dislocation. These findings should be considered cautiously due to potential confounding factors.

### Competing interests

The authors have no conflicts of interest to declare.

### Authors’ contributions

All authors contributed to the conception and design of the study. **LAS** and **MFAR** performed material preparation, literature review, data collection, and quality assessment. **JS** performed the statistical analysis. **LAS**, **HK**, **WA**, **JS**, and **OZA** wrote the first draft of the manuscript and all authors commented on previous versions of the manuscript. All authors read and approved the final manuscript.

## Figures and Tables

**Table 1.** Summary of baseline study characteristics.

Reference	Design, LoE	Country	No. of hips	THA type	FU (years)	Age (years)	Gender (M:F)	Primary diagnosis	Femoral stem types	Bearings	Data source

Furnes et al. (1997)^13^	III, Retrospective	Norway	1127	Boneloc cemented hips	5	72	NA	OA, rheumatoid arthritis (RA)	DePuy, Leeds, England	NA	Norwegian Arthroplasty Register

Cook et al. (2008)^14^	II, Retrospective	Scotland	6458	Cemented	17	67.1	1,385:4,097	NA	Charnley1 (DePuy, Leeds, UK) and ExeterTM (Stryker Howmedica Osteonics, Berkshire, UK)	NA	Royal Infirmary of Edinburgh

Espehaug et al. (2009)^7^	III, Retrospective	Norway	62305	Cemented, hybrid	11	73	28% male	OA	Charnley, Exeter, Titan, Spectron/ITH, Link IP/Lubinus SP	MoP, all poly	Norwegian Arthroplasty Register

Junnila et al. (2016)^15^	III, Retrospective	Finland	286946	Cemented, hybrid	7.4	72	64% female	OA, RA, AVN, DDH, SCFE	CB (Charnley, Lubinus, SpectronEF, Elite), CPT (Exeter, MS-30, C-stem)	NA	Nordic Arthroplasty Register Association (NARA)

Palan et al. (2016)^16^	III, Retrospective	England	257 202	Cemented, hybrid	3.8	73	90,423:166,779	OA	Exeter V40, C-stem, CPT, Charnley	NA	NJR

Scott et al. (2018)^17^	III, Retrospective	USA	1460	Cemented, hybrid	21.7	70	466:994	OA, RA, AVN, DDH, SCFE	CB (VerSys Heritage; Zimmer, Warsaw, IN), CPT (Smith and Nephew, Memphis, TN, Stryker, Mahwah, NJ)	NA	Hospital for Special Surgery in NY

Kazi et al. (2019)^6^	III, Retrospective	England	292987	Cemented	4.2	73.6	101,350:191,632	OA	PTS, Exeter V40, most common, CB Charnley	MOM excluded	UK NJR

Jain et al. (2023)^18^	III, Retrospective	England	584	Cemented	NA	79.1	270:314	NA	PTS (Stryker, CPT, C-stem), CB (Charnley)	NA	Multicenter in UK

Rames et al. (2022)^19^	III, Retrospective	USA	1306	Hybrid	6.1	75.8	431:875	OA	Exeter stem (Stryker, Mahwah, NJ), CB Stryker	Highly cross-linked poly	Mayo Clinic

Heijnens et al. (2023)^20^	III, Retrospective	The Netherlands	76281	Primary	5.1 (SD 3.1)	CPT 74 (SD 8.4) AS 75 (SD 7.3)	CPT 4,490:11,136 AS 15,657:44,998	OA, dysplasia, post-traumatic, osteonecrosis, and others	CPT: Exeter (Stryker), Taperloc (Zimmer Biomet), Twinsys stem (Mathys), C-stem AMT (Johnson & Johnson), AS: Lubinus SPII (Link), Müller (OHST), Stanmore (Zimmer Biomet), Spectron EF (Smith & Nephew)	Metal on cross-linked polyethylene (XLPE), 32,067 (42%) ceramic on XLPE, 27,091 (35.5%)	Dutch Arthroplasty Register (LROI)

White-Gibson et al. (2024)21	II, Prospective	Ireland	1315	Cemented	10		643:708	OA	DePuy Charnley, Stryker Exeter	NA	National Orthopedic Hospital


LoE: level of evidence, FU: follow-up, AVN: avascular necrosis, DDH: hip dysplasia, SCFE: slipped capital femoral epiphysis, AS: anatomically shaped.

**Table 2.** MINORS risk of bias assessment tool used to evaluate included studies.

Reference	A clearly stated aim	Inclusion of consecutive patients	Prospective collection of data	Endpoints appropriate to the aim of the study	Unbiased assessment of the study endpoints	Follow-up period appropriate to the aim of the study

Furnes et al. (1997)^13^	1	1	0	2	0	1

Cook et al. (2008)^14^	2	2	0	2	0	2

Espehaug et al. (2009)^7^	1	1	0	2	0	2

Jayasuriya et al. (2013)^22^	2	2	2	2	2	2

Junnila et al. (2016)^15^	2	1	0	2	0	2

Palan et al. (2016)^16^	2	2	2	2	0	2

Scott et al. (2018)^17^	2	2	2	2	0	1

Kazi et al. (2019)^6^	2	2	2	2	0	2

Jain et al. (2023)^18^	2	2	0	2	0	0

Rames et al. (2022)^19^	2	2	2	2	0	2

Heijnens et al. (2023)^20^	2	2	0	2	0	2

**Table:** 

**Loss to follow-up less than 5%**	**Prospective calculation of the study size**	**Adequate control group**	**Contemporary groups**	**Baseline equivalence of groups**	**Adequate statistical analyses**	**Total**
2	0	2	2	2	2	15
2	0	2	2	2	2	18
2	0	2	2	2	2	16
2	2	2	2	2	2	24
2	0	2	2	2	2	17
2	0	2	2	2	2	20
2	0	1	2	2	2	18
2	0	2	2	2	2	20
0	0	2	2	2	2	14
0	0	2	2	2	2	18
1	0	2	2	2	2	17
